# Association between maternal use of traditional healer services and child vaccination coverage in Pont-Sonde, Haiti

**DOI:** 10.1186/1475-9276-8-1

**Published:** 2009-01-08

**Authors:** Adamson S Muula, Martine Y Polycarpe, Jayakaran Job, Seter Siziya, Emmanuel Rudatsikira

**Affiliations:** 1 Department of Community Health, University of Malawi, College of Medicine, Blantyre, Malawi; 2 Department of Global Health, Loma Linda University, School of Public Health, Loma Linda, California, USA; 3 Department of Community Medicine, University of Zambia, School of Medicine, Lusaka, Zambia; 4 Departments of Epidemiology and Biostatistics and Global Health, Loma Linda University, School of Public Health, Loma Linda, California, USA

## Abstract

**Background:**

Child vaccination is one of the public health interventions that are responsible for the relatively low child morbidity and mortality in developed nations compared to the developing world. We carried out this study to examine the association between mothers' use of traditional healer services and vaccination among Haitian children. Our hypothesis was that children whose mothers used the services of traditional healers were less likely to be vaccinated compared to children whose mothers did not use the services of traditional healers.

**Methods:**

A two-stage stratified sampling method was used to select 720 mothers from the population of Pont-Sonde, Haiti. Of these mothers, 691 (96%) completed the survey by responding to a standardized questionnaire on vaccination giving unadjusted odds ratios (OR) and adjusted odds ratios (AOR) and 95% confidence intervals (CI) and use of traditional healers. Bivariate and multivariate logistic regression analyses were performed to estimate the effect of explanatory variables on vaccination (the main outcome).

**Results:**

Mother's use of traditional healer services was negatively associated with vaccination after controlling for maternal age, education, religion, and distance from the nearest health care facility. For those children whose mothers often or always used the services of traditional healers, we found a 53% decrease in the odds of vaccination (AOR = 0.47; 95% CI [0.27, 0.83]) compared against children whose mothers never used the services of the traditional healers. There were negative associations between practice of Vodou and vaccination (AOR = 0.56; 95% CI [0.35, 0.92]), and distance from the nearest health care service facility and vaccination (AOR = 0.53; 95% CI [0.29, 0.97] and AOR = 0.34; 95% CI [0.20, 0.59] at 46–60 and more than 60 minutes walk time, respectively).

**Conclusion:**

We found that mother's use of traditional healer services was negatively associated with vaccination of Haitian children. Findings from this study underscore the potential to enlist the support of traditional healers in promoting child health by educating, mentoring them (the traditional healers) in supporting vaccination efforts.

## Background

In developing countries, traditional healers often have the role of being the primary health care providers for their communities [1-3]. In addition to traditional birth attendants (TBAs), there are distinct groupings of traditional healers that provide primary health care in communities, in different forms based on their skill level, their accessibility, and whether they underwent lengthy apprenticeships or a spiritual "calling" to their role [4-6].

Although many traditional healers are herbalists, this is not the only way traditional health care is practiced. Some call upon the ancestral spirits or perform exorcism to treat an illness, yet the herbalist may also incorporate this spiritual aspect in diagnosing the patient's illness [3,5,7]. Faith healers may utilize prayer, touch, and ointments in their healing rituals. There are also healers who combine Islamic medicine, and will invoke verses of the Koran and/or use astrology in the healing process [3].

The services of a traditional healer may be sought in cases where the illness is considered 'native' in origin. However, if the illness is considered to have a foreign origin, a Western medical provider may be consulted, out of the belief that they will be more familiar and have better treatment. The treatment is determined as specifically relating to the source of the illness, whether worldly or other-worldly. The use of traditional healers is widespread and accepted in most Haitian communities. Twenty-three percent of mothers go to traditional healers when their children are sick [8].

A Presidential decree of April 4, 2003 recognized Vodou as a bonafide religion in Haiti. With regard to the decree, Vodou chiefs, temple officials, officials at any sacred site, as well as all Vodou organizations or associations were empowered to file a request for recognition by the Ministry of Culture and Religious Affairs. It is possible for people in Haiti to belong to a mission religion such as Catholicism or Protestant Christianity but still practice Vodou [9,10].

According to the World Health Organization (WHO), the two public health interventions that have had the greatest impact of the world's child health are clean water and vaccines. In light of this fact the importance of encouraging vaccination in Haiti is obvious benefit. Vaccinations against tuberculosis, diphtheria, pertussis, tetanus, measles, and polio have dramatically reduced the burden of death and disease from infectious diseases [11,12]. However, vaccination services continue to be under-utilized, especially in developing nations. Almost two million children still die each year from vaccine-preventable infectious diseases [13]. In Haiti, more than 138,000 children under five years of age die of preventable diseases annually; 60% of those who survive may fail to develop adequately [14]. The major causes of mortality among children under five years old are diarrhea, malnutrition, measles and malaria. Furthermore, because measles enhances deficiency in vitamin A, it continues to be an important cause of blindness [15]. Only 30% of Haitian children 12–23 month old are fully vaccinated. There appears to be no sex or urban versus rural differences in vaccination prevalence in the country [8].

Over the years, the World Health Assembly has adopted a number of resolutions encouraging the study of the potential usefulness of traditional medicine including evaluation of practices and examination of safety and efficacy of remedies used. The WHO also advocates for the education and information dissemination of the public about proven traditional health practices [16-18]. Globally, traditional healers have been reported to offer treatments for hypertension, [19] cancer, [20] AIDS, [21] tuberculosis, [22,23] malaria, [3,24,25] sexually transmitted infections, [26] epilepsy, [27] and infertility [28]. However, there is paucity of studies on the role of traditional healers in vaccination. The purpose of this study was to examine the association between the use of traditional healers by mothers and child vaccination.

## Methods

### Study sample design and recruitment

To enroll our sample we conducted a two-stage cluster sampling of the residents of the town of Pont-Sonde, Haiti – a town with approximately 20,000 residents. In the first stage of sampling, we randomly sampled administrative sub-units of the town. In the second stage of sampling we randomly selected houses from each administrative sub-unit. Mothers (18 years or older) of children five years or younger in selected households were eligible to participate in the study. Mothers were excluded if they were younger than 18 years or had no child 5 years old or younger. Trained interviewers administered the questionnaire to mothers. They asked mothers to show them the child vaccination card to validate vaccination records. The data were collected during the first three weeks of September 2003. Interviewers visited the homes in the afternoon or evening when mothers had returned from the fields or markets.

### Questionnaire administration

The study's instrument was a modified version of the Knowledge, Practice, and Coverage survey questionnaire used in the US Child Survival Technical Support Project [29]. The survey instrument consisted of 44 questions, with eleven questions on demographic variables and 33 questions on health-seeking behaviors, vaccination status of children in the household, education level, breast feeding practices and other maternal/child health factors that are conducive to disease prevention. Trained interviewers administered the questionnaire in approximately 40 minutes. Child vaccination status was determined through inspection of a child's vaccination card by the study interview. Maternal religious affiliation, education and practice of traditional healing information were obtained from self-identified and reporting by the mother. For the purpose of this study however, maternal religious denomination was restricted to the one preferred and self-identified by the mother.

### Statistical methods

We calculated the frequencies and proportions of: age groups; maternal education; religion; child vaccination status and use of traditional healers. We used a logistic regression model considering "fully vaccinated status" as our dependent variable, and the following independent variables: use of traditional healers, age, education, religion, and distance from the nearest health care facility. We report both unadjusted odds ratios (OR) and adjusted odds ratios (AOR) and 95% confidence intervals (CI). Fully vaccinated status was defined as having had the following vaccines: BCG vaccine, Polio 3, measles, and Diphteria-Pertussis 3 and Tetanus. We accounted for the two-stage sampling design in our variance calculations of the odds ratios. All data analyses were conducted using SUDAAN version 9.0 (Research Triangle Institute, Research Triangle, Durham, North Carolina).

### Human subjects

The Institutional Review Board (IRB) of Loma Linda University approved the study protocol. Informed consent was obtained from study participants.

## Results

Of the 720 eligible mothers, 691 (96%) completed the survey. Table 1 presents selected characteristics of the study population (mean age 28.9 years, standard deviation 10.1 years). Most of the respondents were in the age group 25–34 years old (42.6%), and had elementary education (52.5%).

**Table 1:** Selected demographics and vaccination coverage among Haitian children five years and under

	N	% [95%CI]
**Demographics of mothers**		

Age (years)	691	100.0

<25	234	34.2 [32.2, 36.2]

25–34	299	42.6 [40.4, 44.8]

35–44	131	19.4 [17.7, 21.2]

>44	27	3.9 [3.2, 4.7]

Education	675	100.0

No formal education	171	24.1 [20.7, 27.9]

Elementary	361	52.5 [48.1, 56.8]

Secondary	143	23.5 [19.9, 27.4]

Religion	683	100.0

Protestant	243	34.0 [30.0, 38.2]

Catholic	224	39.9 [29.9, 38.1]

Vodoo	216	32.1 [28.2, 36.3]

**Distance to hospital or health center (minutes)**	673	100.0

0–15	116	17.2 [14.9, 21.7]

16–30	148	22.0 [17.1, 28.4]

31–45	140	20.8 [16.8, 25.7]

46–60	73	10.9 [6.5, 14.8]

> 60	196	29.1 [25.3, 34.8]

**Child vaccination**		

BCG	615/648	93.6 [90.9, 95.5]

Polio 1 and Diptheria-Pertussis and Tetanus 1	563/655	83.9 [80.3, 86.9]

Polio 3 and Diptheria-Pertussis and Tetanus 3	420/653	63.1 [58.7, 67.3]

Measles	330/657	51.3 [46.9, 55.7]

Fully immunized	319/683	47.0 [42.7, 51.3]

**Use of traditional healer's services**	691	100.0

Often or always	120	16.6 [13.6, 20.0]

Sometimes	233	31.7 [27.9, 35.9]

Never	336	51.7 [47.4, 56.0]

Those who were affiliated to Vodou were 32.1% of the sample. Nine in ten children (93.6%) had BCG (Bacille Calmette-Guérin), the vaccination against tuberculosis and one in two had measles vaccination (51.3%). Only 47% of children five years of age or younger were fully vaccinated. About one half of mothers reported using the services of traditional healers (48.3%).

### Variables associated with vaccination

The data from table 2 bivariate results indicate that use of traditional healers was inversely associated with child vaccination. Children whose mothers often or always used traditional healers had a 62% decrease of being fully vaccinated compared to those whose mothers never used traditional healers (OR = 0.38; 95% CI [0.23, 0.65]. Those whose mothers sometimes used traditional healers had a 37% decrease in the odds of fully immunized (OR = 0.63; 95% CI [0.43, 0.94]). Vodou and Catholicism were associated with 54% and 39% decrease in the odds of being fully vaccinated (OR = 0.46; 95% CI [0.30, 0.71] and OR = 0.61; 95% CI [0.39, 0.94], respectively). Living at a more than one hour walking distance from the nearest health care facility was associated with a 66% decrease in the odds of being fully vaccinated (OR = 0.34; 95% CI [0.20, 0.58]). Mothers' education was not significantly associated with child vaccination.

**Table 2:** Maternal characteristics associated with vaccination among Haitian children five years and under in bivariate analyses

**Age (years)**	**Adjusted odds ratios with 95%CI**
<25	1.00

25–34	1.18 [0.78, 1.73]

35–44	1.14 [0.69, 1.88]

>44	0.81 [0.32, 2.07]

**Education**	

Secondary	1.00

Elementary	0.90 [0.58, 1.40]

No formal education	0.67 [0.40, 1.11]

**Religion**	

Protestant	1.00

Catholic	0.61 [0.39, 0.94]

Vodou	0.46 [0.30, 0.71]

**Distance to hospital or health center (minutes)**	

0–15	1.00

16–30	0.81 [0.45, 1.45]

31–45	0.57 [0.32, 1.00]

46–60	0.54 [0.27, 1.08]

> 60	0.34 [0.20, 0.58]
**Use of traditional healers**	

Never	1.00

Sometimes	0.63 [0.43, 0.94]

Often or always	0.38 [0.23, 0.65]

Table 3 indicates that use of traditional healers was negatively associated with child vaccination even after controlling for age, education, religion, and distance from the nearest health care facility. Children whose mothers often or always used traditional healers had a 53% decrease in the odds of being fully vaccinated compared to children whose mothers never used traditional healers (AOR = 0.47; 95% CI [0.27, 0.83]). Using traditional healers sometimes was associated with 41% decrease in the odds of vaccination (AOR = 0.59; 95% CI [0.39, 0.90]). Affiliation to Vodou, and living at distances that took 46–60 minutes or more than 60 minutes walking time from the nearest health care facility were negatively associated with vaccination (AOR = 0.56; 95% CI [0.35, 0.92], AOR = 0.53; 95% CI [0.29, 0.97], and AOR = 0.34; 95%CI [0.20, 0.59], respectively).

**Table 3:** Maternal characteristics associated with vaccination among Haitian children five years and under in multivariate analysis

**Age (years)**	**Odds ratios with 95% CI**
<25	1.00

25–34	1.19 [0.77, 1.83]

35–44	1.34 [0.76, 2.35]

>44	1.05 [0.39, 2.85]

**Education**	

Secondary	1.00

Elementary	0.86 [0.49, 1.53]

No formal education	0.80 [0.49, 1.31]

**Religion**	

Protestant	1.00

Catholic	0.74 [0.47, 1.18]

Vodou	0.56 [0.35, 0.92]

**Distance to hospital or health center (minutes)**	

0–15	1.00

16–30	0.94 [0.55, 1.60]

31–45	0.68 [0.7, 1.27]

46–60	0.53 [0.29, 0.97]

>60	0.34 [0.20,0.59]

**Use of traditional healers**	

Never	1.00

Sometimes	0.59 [0.39, 0.90]

Often or always	0.47 [0.27, 0.83]

## Discussion

In this study, we found that 47% of the subjects were fully vaccinated, which is a slightly higher than the national average of 43%. Vaccination coverage in Haiti is far below the 78% global vaccination coverage [30] and the 88% vaccination coverage of the Pan American Health Organization (PAHO) region [31].

The use of traditional healers was negatively associated with vaccination. This may be an indication that traditional healers do not support the use of the formal health sector which includes vaccination. Burnet and Buggaley [32] report that there is often mistrust and lack of understanding between traditional healers and the formal health sector, but traditional healers are more open for collaboration than formal health workers. Several studies have indicated that collaboration between traditional healers and the formal health care sector is possible, useful and crucial in order to bridge the treatment gap in developing countries [25,33-36].

Vodou affiliation was associated with decreased vaccination rate, which may be an indication that Vodou practice may not be favorable to vaccinations. It is also possible that Vodou affiliation may just be an indicator of some unmeasured variable which may also be associated with non-vaccination of children.

The United Nations Children Fund (UNICEF) recommends that public health workers collaborate with religious leaders and traditional healers whose support can help reach out to members of the community who are reluctant to vaccinations [37]. It is encouraging to note that a number of modern health care groups have already started working with Vodou priests in Haiti to help identify people who are HIV-positive [38]. While this recommendation is appropriate, only a future evaluation will show or fail to show if collaboration with Vodou priests will improve the prevalence of children who will be fully vaccinated.

Our findings indicate that the proximity to a health care facility was positively associated with full vaccination. Child vaccinations are provided for free at the clinic in Pont-Sonde and at the nearest hospital. Previous studies have identified long distances from vaccination site as a risk factor for failure to be vaccinated [39,40]. Reichler et al [39] reported that in Egypt, being farther than 10 minutes by foot from the nearest National Immunization Day (NID) site was a risk factor for failing to receive NID vaccines. In Kenya, Ndirutu et al [40] found that vaccination coverage was reduced with every kilometer of distance from home to vaccination clinic. In Haiti it might be worthwhile to facilitate intermittent vaccination campaigns within the community which may reduce the distance from homes to vaccination sites.

In this study, maternal education was not associated with child vaccination. At least three-quarters of women in our sample had elementary education only or none. Unlike in other settings where women with low education or none may be worse off than others, we find that there was not a difference in vaccination status based on maternal education. This may have been that there was limited variability in resources (cognitive or material) that may have influenced decision to vaccinate e.g. the knowledge, attitudes and behaviors which influence decisions to vaccinate may have been similar and educated women did not have better financial resources to facilitate transport to clinic [41,42].

This study had several limitations. Firstly, this was a cross-sectional study and we, therefore, cannot assume causality i.e. none of the factors associated (positively or negatively) with vaccination can be taken as have caused the vaccination. Secondly, some participants may have mis-reported their use of traditional healers. Although the use of traditional healers is widely accepted in Haiti, some medical professionals discourage or have negative attitudes towards the practice. This may have led some mothers to underreport their use of traditional health services; in such case, the reported odds ratios would be conservative values. Furthermore, we categorized Vodou affiliation mutually exclusive with other religions. To the extent that mothers affiliation to Vodou and other religions, our results may be biased. However, such bias may be that the measure of association (the OR) would tend to move towards the null (equality). This would mean that the effects that we have estimated are in fact under-estimates.

## Conclusion

Among Haitian children, we found that mother's use of traditional healers was negatively associated with vaccinations. Findings from this study underscore the need to undertake education efforts towards traditional healers and to include them in vaccination efforts. Given the success other areas of modern health care have had in working with traditional healers, including vaccination programs in that collaboration could considerably increase vaccination coverage in Haiti. This approach may also be explored in other developing countries experiencing low vaccination coverage.

## Competing interests

The authors declare that they have no competing interests.

## Authors' contributions

ER participated in the study design, conducted data analysis and participated in the drafting of manuscript. MYP participated in study design, conducted data collected and participated in the drafting of manuscript. JJ participated in study design, interpretation of findings and drafting of manuscript. SS participated in the interpretation of findings and drafting of the manuscript. ASM led the manuscript drafting effort and participated in the interpretation of findings. All author approved the final draft of the manuscript.

## References

[B1] Chipfakacha V (1994). The role of culture in primary health care (Two case studies). S Afr Med J.

[B2] Chana H, Schwab L, Foster L (1993). With an eye to good practice: Traditional Healers in rural communities. World Health Forum.

[B3] Makemba AM, Winch PJ, Makame VM, Mehl GL, Premji Z, Minjas JN, Shiff CJ (1996). Treatment practices for *degedege*, a locally recognized febrile illness, and implications for strategies to decrease mortality from severe malaria in Bagamoyo District, Tanzania. Trop Med Int Health.

[B4] Silkkerveer LJ (1990). Plural medical systems in the Horn of Africa.

[B5] Kale R (1995). Traditional healers in South Africa: a parallel healthcare system. BMJ.

[B6] Hewson MG (1998). Traditional Healers in Southern Africa. Ann Intern Med.

[B7] Freeman M, Motsei M (1992). Planning Health Care in South Africa-Is There A Role For Traditional Healers?. Soc Sci Med.

[B8] Cayamettes M, Placide MF, Barrere B, Soumaila M, Severe B (2000). Enquete mortalite, morbidite et utilization des services, Haiti 2000.

[B9] Nicholls D (1970). Politics and religion in Haiti. Can J Polit Sci.

[B10] Largey M (2006). Vodou: Haitian art music and cultural nationalism.

[B11] World Health Organization The history of vaccination. Vaccines, Immunization and Biologicals: Diseases and Vaccines. http://www.who.int/vaccines-diseases/history/history.shtml.

[B12] Orenstein WA (2006). The role of measles elimination in development of a national immunization program. Pediatr Infect Dis J.

[B13] World Health Organization The world's forgotten children. http://www.who.int/entity/ceh/publications/.

[B14] ORC and Institute for Resource Development (2000). Haiti Demographic and Health Survey 2000.

[B15] Titiyal JS, Pal N, Murthy GV, Gupta SK, Tandon R, Vajpayee RB, Gilbert CE (2003). Causes and trends of blindness and severe visual impairment on children in schools for the blind in North India. Br J Ophthalmol.

[B16] World Health Organization (1997). Thirtieth World Health Assembly. A30/A/SR/18.

[B17] World Health Organization (1978). Report of the International Conference on primary health care.

[B18] World Health Organization (1989). Forty-second World Health Assembly. WHA 4243.

[B19] Abel C, Busic K (2005). An explanatory ethnobotanical study of the practice of herbal medicine by the Akan peoples of Ghana. Altern Med Rev.

[B20] Ariffin H, Abdullah WA, de Bruyne J, Lee CL, Peng LH (1997). Beliefs in traditional healers among Malaysian parents of children with cancer. J Trop Pediatr.

[B21] Green EC (2000). Traditional healers and AIDS in Uganda. J Altern Complement Med.

[B22] Wilkinson D, Gcabashe L, Lurie M (1999). Traditional healers as tuberculosis treatment supervisors: precedent and potential. Int J Tuberc Lung Dis.

[B23] Brouwer JA, Boeree MJ, Kager P, Varkevisser CM, Harries AD (1998). Traditional healers and pulmonary tuberculosis in Malawi. Int J Tuberc Lung Dis.

[B24] Randrianarivelojosia M, Rasidimanana VT, Rabarison H, Cheplogoi PK, Ratsimbason M, Mulholland DA, Mauclere R (2003). Plants and traditionally prescribved to treat tazo (malaria) in the eastern region of Madagascar. Malaria J.

[B25] Makundi EA, Malebo HM, Mhame P, Warsame AM (2006). Role of traditional healers in the management of severe malaria among children below five years of age: the case of Kilosa and Handeni Districts, Tanzania. Malaria J.

[B26] Ndubani P, Hojer B (1999). Traditional healers and the treatment of sexually transmitted illnesses in rural Zambia. J Ethnopharmacol.

[B27] Basking R, Birbeck G (2005). Epilepsy care in Zambia: A study of traditional healers. Epilepsia.

[B28] Obisesan KA, Adeyemo AA (1998). Infertility and other fertility related issues in the practice of traditional healers and Christian religious healers in south western Nigeria. Afr J Med Med Sci.

[B29] United States Child Survival Technical Support Project KPC 2000 + Field Guide. http://www.childsurvival.com.

[B30] World Health Organization Immunization surveillance, assessment and monitoring. http://www.who.int/vaccines/globalsummary/immunization/timeseries/tscoveragemcv.htm.

[B31] Pan American Health Organization Vaccines and immunization. http://devserver.paho.org/hq/index.php?option=com_content&task=view&id=287&Itemid=381.

[B32] Burnett A, Baggaley R (1999). Caring for people with HIV in Zambia: are traditional healers and formal health workers willing to work together?. AIDS Care.

[B33] Poudyal AK, Jimba M, Poudyal BK, Wakai S (2005). Traditional healers' roles on eye care services in Nepal. Br J Ophthalmol.

[B34] Zachariah R, Nkhoma W, Harries AD, Arendt V, Chantulo A, Spielmann MP, Mbereko MP, Buhendwa L (2002). Health seeking and sexual behaviour in patients with sexually transmitted infections: the importance of traditional healers in Thyolo, Malawi. Sex Transm Infect.

[B35] Hoff W (1992). Traditional healers and community health. World Health Forum.

[B36] Courtright P, Charambo M, Lewallen S (2000). Collaboration with African traditional healers for the prevention of blindness.

[B37] United Nations Children Fund Traditional healers and community. http://www.ncbi.nlm.nih.gov/entrez/query.fcgi?cmd=Retrieve&db=PubMed&list_uids=1.

[B38] Fort S Paying respect to traditional medicine: modern health care groups collaborate with vodoo priest in Haiti to help identify people who are HIV-positive.

[B39] Reichler MR, Darwish A, Stroh G, Stevensen J, Al Nasar MA, Oun SA, Wahdan MH (1998). Cluster survey evaluation of coverage and risk factors for failure to be immunized during the 1995 National Immunization Days in Egypt. Int J Epidemiol.

[B40] Ndirutu M, Cowgill KD, Ismail A, Chiphatsi S, Kamau T, Fegan G, Feikin DR, Newton CRJC, Scott AG (2006). Immunization coverage and risk factors for failure to immunize within the Expanded Programme on Immunization in Kenya after the introduction of new Haempphilus influenzae type b and hepatitis b virus antigens. BMC Public Health.

[B41] Adler NE, Newman K (2002). Socioeconomic disparities in health: pathways and policies. Health Aff.

[B42] Adler NE, Ostrove JM (1999). Socioeconomic status and health: what we know and what we don't know. Ann N Y Acad Sci.

